# P-979. DASC-LOT Framework: A Novel Evaluation and Benchmarking Method to Assess Initiation, Duration, and Spectrum of Antibiotics Usage at Hospitals

**DOI:** 10.1093/ofid/ofaf695.1178

**Published:** 2026-01-11

**Authors:** Michihiko Goto, James Merchant, Hyunkeun Cho, Matthew B Goetz, Daniel J Livorsi

**Affiliations:** University of Iowa/Iowa City VAMC, Iowa City, IA; Iowa City VA Health Care System, Iowa City, Iowa; University of Iowa Carver College of Medicine, Iowa City, Iowa; VA Greater Los Angeles Healthcare System, Los Angeles, California; University of Iowa Carver College of Medicine, Iowa City, Iowa

## Abstract

**Background:**

Inpatient antibiotic stewardship programs (ASP) promote avoiding unnecessary initiation, excessively long duration, and overly broad-spectrum selection of antibiotics to optimize usage. Commonly used metrics, such as Days of Therapy (DOT) per Days Present (DP) or Standardized Antimicrobial Administration Ratio (SAAR), reflect the overall usage but do not incorporate spectrum, nor provide specific information for components (initiation, duration, and spectrum). We aimed to create a novel framework to reflect all three components while providing information specific to each, based on Days of Antimicrobial Spectrum Coverage (DASC).

**Figure 1.**
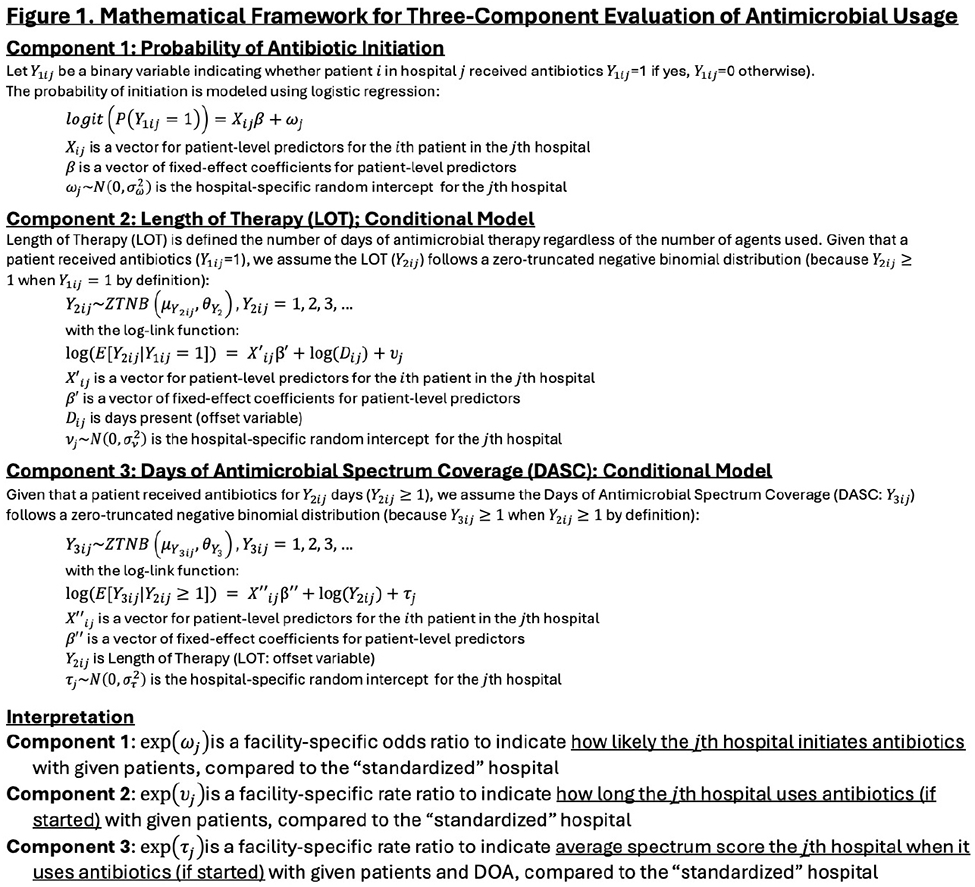
Mathematical Framework for Three-Component Evaluation of Antimicrobial Usage

**Figure 2.**
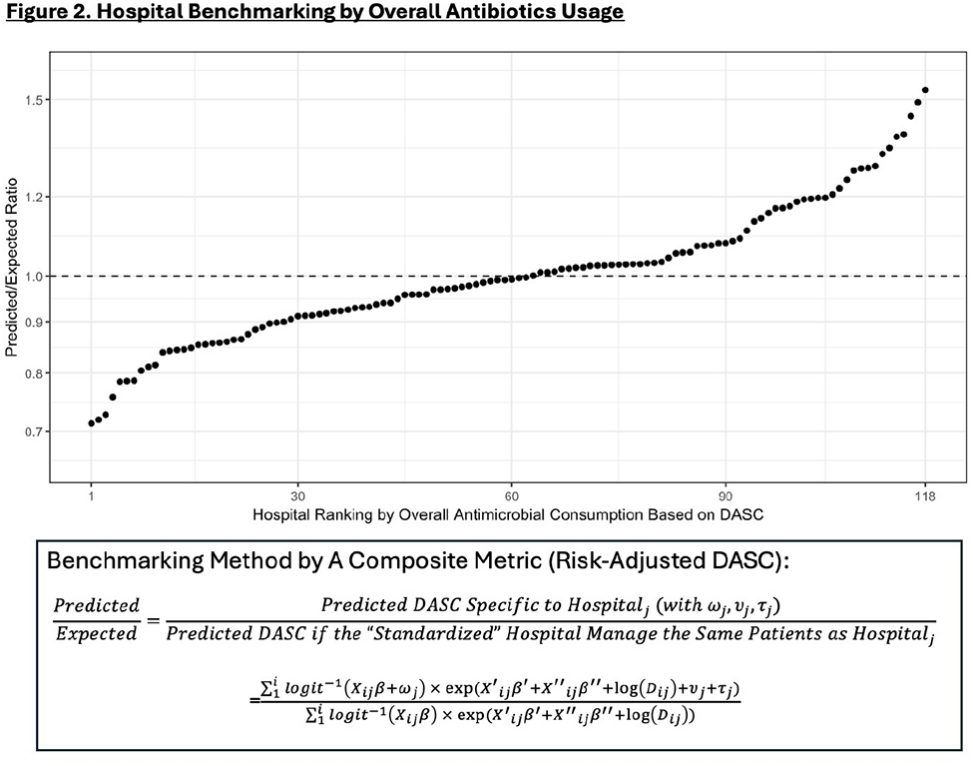
Hospital Benchmarking by Overall Antibiotics Usage

**Methods:**

We developed a mathematical framework to extract hospital-level variability with risk adjustment for three components (Figure 1), using DASC and length of therapy (LOT). This was applied to data from all 118 Veterans Health Administration (VHA) acute care hospitals, with models built on 2022–2023 data and validated with 2024 data. Patient demographics, intensive care status, specialty, 86 comorbidities, and 225 procedure categories were considered as candidate variables for risk-adjustments. Overall hospital performances were evaluated by composite metrics (predicted/expected [P/E] ratio), integrating three components (Figure 2), and three components in each hospital were visualized in a radar chart (Figure 3).

**Figure 3.**
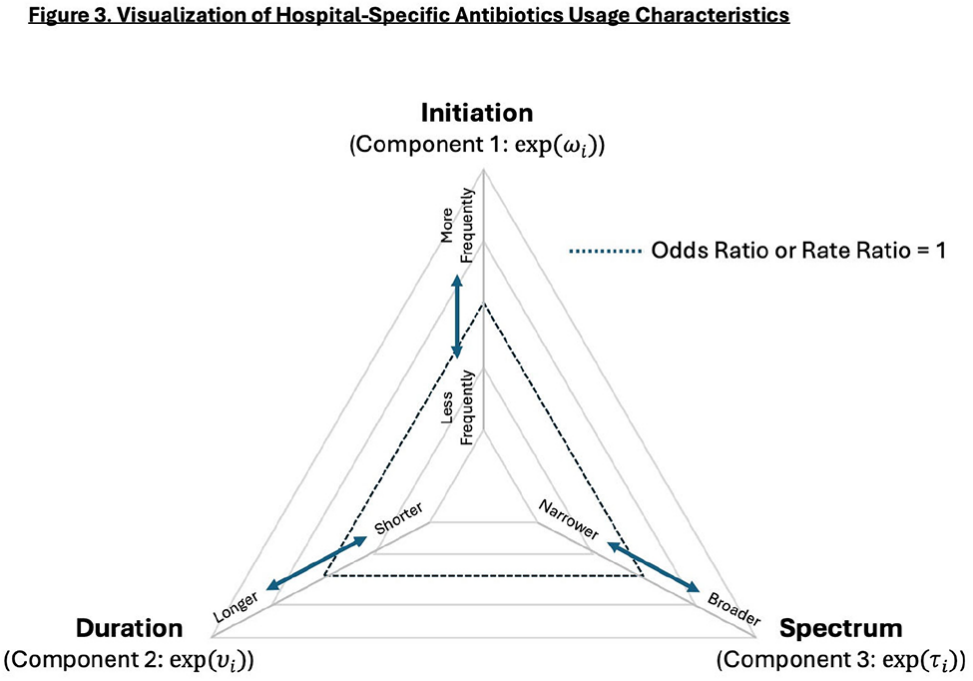
Visualization of Hospital-Specific Antibiotics Usage Characteristics

**Figure 4.**
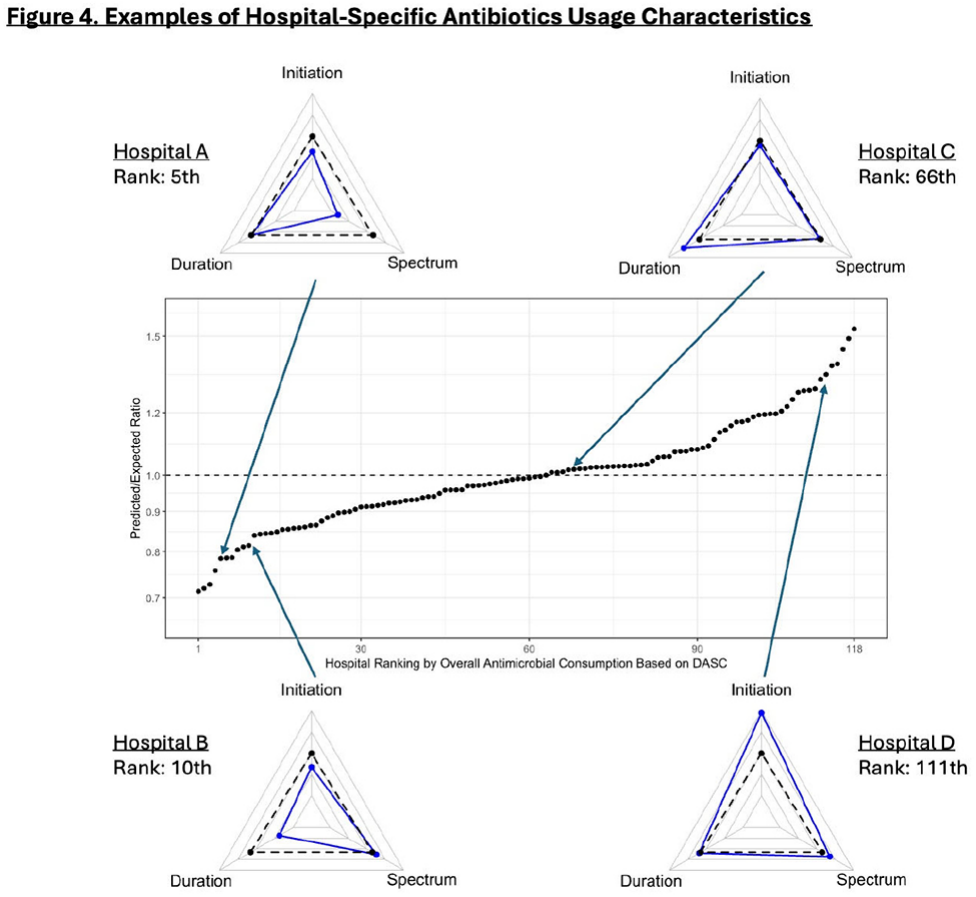
Examples of Hospital-Specific Antibiotics Usage Characteristics

**Results:**

The cohort included 727,958 unique patients with 9,363,922 days present (DP: 2022-2023: 6,257,368; 2024: 3,106,554). Hospital-level usage density ranged widely (DASC per 1,000 DP: 1,311-5,275 [interquartile range (IQR): 2,738-3,563]; LOT per 1,000 DP: 132.2-517.6 [IQR: 301.0-367.5]). Risk-adjustment models included 115 variables for initiation, 125 for duration, and 128 for spectrum components. P/E ratios ranged from 0.713 to 1.533 [IQR: 0.912-1.077] (Figure 2). Three-component evaluation could offer more specific information for each hospital about its usage pattern (Figure 4).

**Conclusion:**

We propose a novel framework to assess ASP practices in initiation, duration, and spectrum separately while providing overall composite benchmarking. Further studies are needed to assess whether this framework reflects the appropriateness of antibiotic therapies or outcomes.

**Disclosures:**

All Authors: No reported disclosures

